# The Oxygen Treatment of Ulcers—An Anonymous Symposium

**Published:** 1897-03-13

**Authors:** 


### THE OXYGEN TREATMENT OF ULCERS—AN ANONYMOUS SYMPOSIUM.

In view of the importance of the scientific and ethical questions involved in the establishment of a special institute for the treatment of ulcers by means of oxygen, we addressed a letter to a few well-known surgeons connected with the great school hospitals of the metropolis asking for their opinion (1) as to the utility of the proceeding, and (2) as to the desirability of establishing a separate institute for its performance.

We received a considerable number of answers to our inquiries, the majority of which, however, were unfortunately marked "private." We are thus precluded from publishing a pronouncement on the subject which would have been both interesting and authoritative, all the writers being well-known men, occupying influential positions in the hospital world. Under the circumstances, it seems to us that the best thing we can do is to analyse these communications and publish their main purport in the form of an anonymous symposium.

In regard to the utility of oxygen in the healing of ulcers, we meet with very little in these letters to corroborate the statements made by Dr. Stoker. It should at the same time be said that a great number of the writers admit that they have had but small, and, in many cases, no personal experience on the subject.

Mr. Marmaduke Sheild says that he has seen enough of Dr. Stoker's cases to convince him that oxygen gas, when aided by rest and cleanliness, markedly promotes the healing of ulcers. How far it is superior to other methods, such as irrigation and well-conducted aseptic skin grafting, he is not prepared to say. The influence of gases on micro-organisms is a large and complicated subject, and he looks on the action of staphylococci as "rather nebulous." According to Dr. Stoker they seem to flourish in oxygen, but it seems far from certain that they have anything to do with the stimulation of epithelial growth. Nevertheless, he thinks that the subject is, without doubt, one to engage the attention of bacteriological experts.

The latter point is one in which several of the writers are agreed. Mr. Henry Morris thinks that it should be scientifically tested in the laboratory in reference to the behaviour of the staphylococcus under different atmospheres. Another writer feels strongly that the oxygen treatment ought to be tried at the general hospitals, and, if found successful, ought to become part of ordinary routine treatments at the surgeon's disposal. He says that the bacteriological point is a most important one, and certainly ought to be confirmed or refuted; and he points out that, although experiments could be made in the laboratory to show the influence of oxygen on the growth of staphylococci, the question could only be settled, and Dr. Stoker's point verified, by a bacteriologist working at a hospital where the treatment was carried out.

Several writers do not feel inclined to express a positive opinion on the suggested treatment, but many express the opinion very definitely that the thing should be tried and properly threshed out in the general hospitals; and one says that, although a treatment might to him seem useless, he would not like to do anything to stop a man from carrying it out, because in the end there might be something in it, provided, of course, that he saw no indication of dishonesty of purpose. In regard to the bacteriological point several writers state that the cocci in question are known to flourish under the influence of oxygen, and that so long as they grow on healthy granulations, and the barrier is not broken down by probing or any injury, no harm comes from their growth on the surface. No one, however, seems ready to admit that they can do good.

On the other hand some very adverse opinions have been expressed. It is said by some that the inoculation of wounds with pyogenic cocci is absurd and almost criminal. The whole thing is said to show inexcusable ignorance; several writers express some astonishment, and others disgust, that self-respecting societies should admit such cases on exhibition, and several decline to have anything to do with it.

Almost universally the opinion is expressed that, as we suggested in our recent article on the subject, the healing of an ulcer is not a real difficulty when it is taken into a hospital ward and treated properly.

A very interesting clinical point is mentioned by several of our correspondents, namely, that the difficulty is not in the healing of the ulcer, but is due to the tendency it so often shows to recur when the old conditions are renewed. Mr. Henry Morris says, "But speaking generally it is not so much a fresh therapeutic measure for ulcers that is wanted. Ulcers under rest, elevation of the part, and simple dressings, &c., yield readily to treatment. But what would be an inestimable boon to thousands of poor working men and women is a means of keeping the part sound after the ulcer has been healed. Hitherto skin grafting has done most this way, but unhappily, even after skin-grafting an ulcer which has taken six weeks or more to heal, will break down, only too often, after the patient has been on her legs again for as many months."

Another gentleman, who writes to us very fully on the whole subject, points out how seldom chronic ulcers are admitted as in-patients; but says that when they do have such an ulcer in the wards there is not generally any trouble in healing it, even when due to prolonged constitutional disease, or to bad varicose veins, or other such causes. The trouble is not to heal it, but to keep it healed when the patient goes out, resumes prolonged standing occupations, and allows the parts to get into a filthy condition. He asks whether Dr. Stoker claims for the cicatrix produced under oxygen, that it consists of better and firmer cicatrical material, and does not break down under these conditions. If so, he says the assertion ought probably to be tested, but he does not think the mere fact of the ulcer healing worthy of special trial.

Mr. Marmaduke Sheild also refers to the same point, saying that he is not clear as to the permanency of the results; for, although many an ulcer can be readily healed, there is a great tendency to relapse when exposed to the effects of congestion and other evil influences.

With very few exceptions our correspondents agree that in regard to the mere healing of an ulcer there is no excuse for any new treatment, and it is said by some that not only is there no advantage in the oxygen treatment, but that the results as regards ulcers are in no way to be compared with what ought to be obtained nowadays.

Still, we cannot but think that the treatment ought to be properly tried, so that, if of use, its utility may be more widespread, and, if of no use, it may be definitely relegated to oblivion; and we cannot but agree with the words of one of the most distinguished of our correspondents, that "trial in the general ward of a hospital is the best way of obtaining evidence on the subject."

